# Investigation on Composition, Mechanical Properties, and Corrosion Resistance of Mg-0.5Ca-X(Sr, Zr, Sn) Biological Alloy

**DOI:** 10.1155/2018/6519310

**Published:** 2018-04-23

**Authors:** Yichang Su, Jixing Lin, Yingchao Su, Wei Zai, Guangyu Li, Cuie Wen

**Affiliations:** ^1^Key Laboratory of Automobile Materials, Ministry of Education, College of Materials Science and Engineering, Jilin University, Changchun 130025, China; ^2^School of Engineering, RMIT University, Melbourne, VIC 3001, Australia

## Abstract

Four nontoxic biological alloys, Mg-0.5Ca-1Sr-4Zr (Alloy 1), Mg-0.5Ca-1Sr-1.5Zr (Alloy 2), Mg-0.5Ca-3Sr-1.5Zr (Alloy 3), and Mg-0.5Ca-1Sr-0.5Sn (Alloy 4), were prepared by vacuum smelting, gravity casting, and hot rolling. The composition and microstructure of the alloys were investigated by optical microscope, X-ray fluorescence spectrometer (XRF), X-ray diffraction (XRD), scanning electron microscope (SEM), and energy dispersion spectroscopy (EDS). The mechanical properties and corrosion behaviors of the alloys in Hank's solution were studied. Results showed that a large amount of fine and uniformly distributed second-phase particles (Zr, Mg_17_Sr_2_, and CaMgSn) was observed in four alloys obtained after rolling and alloying. The segregation of Zr in alloys was observed in EDS image, and chemical analysis showed that there was macrosegregation of the elements in the alloys. Furthermore, Mg_17_Sr_2_ phases in the Mg-0.5Ca-1Sr-0.5Sn alloy homogenized the distribution of CaMgZn phases. The comprehensive mechanical properties of four newly designed rolled alloys were much higher than those of pure Mg, and the compressive strength of the alloys was more than twice as high as that of pure magnesium. The Mg-0.5Ca-1Sr-0.5Sn alloy released the least hydrogen in Hank's solution, which was lower than that of pure magnesium. Electrochemical test results in Hank's solution further showed that the Mg-0.5Ca-1Sr-0.5Sn alloy had delayed corrosion and lowest *I*_corr_ which was 25% of that of pure magnesium. Biological experiments results showed that the Mg-0.5Ca-1Sr-0.5Sn alloy had better biocompatibility and optimal potential for bone substitute material.

## 1. Introduction

In recent years, titanium alloy, 316L stainless steel [1, 2], and cobalt-based alloys [3, 4] are widely used in bone substitute materials [5–7]. However, poor biocompatibility impacted 316L SS application. Its implantation into the human body may cause the occurrence of allergic reactions to nickel [8]. Ebadian et al. compared various implantation materials and found that the chrome cobalt (Co-Cr) group was more toxic than the other groups; inflammation increased over time [9]. Magnesium and its alloys have been attracting growing attention as next-generation medical material suitable for biodegradable bone implant and stent, due to their well physical and mechanical properties, such as excellent biocompatibility, high strength, and similar Young's modulus to human bone [10–13]. Magnesium and its alloys' density is approximately 1.8–2.0 g·cm^−3^, which is closer to the density of human bone (1.7–2.0 g·cm^−3^). Titanium alloy, 316L stainless steel, and cobalt-based alloy were up to 4.0 g·cm^−3^, 7.9 g·cm^−3^ or more, and 8.3 g·cm^−3^ or more, respectively. Magnesium alloys' Young's modulus is 41–45 GPa, which is closer to that of the human bone density (10–40 GPa), while titanium alloy, 316L stainless steel, and cobalt-based alloy Young's modulus were up to 110 GPa, 189 GPa, and more than 230 GPa [10].

What hindered the development of magnesium alloy was the corrosion resistance of magnesium alloy. As a biocompatible implant material, magnesium alloys were susceptible to corrosion in human body which was rich in Cl^−^ ions, leading to premature failure of its mechanical properties as a key problem for the researchers [14]. In order to improve the corrosion resistance of the biocompatible magnesium alloy, fine-graining [15–17], high-purification [18], alloying [19], and surface treatment [20, 21] were commonly used.

Alloying is a hot topic in improving the comprehensive properties of biological magnesium alloys. Ca is an important constituent element of bone tissue, which can increase the strength and corrosion resistance of the alloy while increasing the solid solubility of Zr in the magnesium alloy and promote the refinement of the alloy microstructure [10, 19, 22]. Zr as an alloying element can significantly refine the grains, enhance the tensile strength and yield strength of the alloy, and in the meantime form the precipitation phase to improve the corrosion resistance of the alloy [10, 23]. Sr can promote the growth of osteoblasts, accelerate the healing of bone tissue, and improve the mechanical properties, corrosion resistance, and high temperature creep resistance of the alloy [24–31]. After rolling, the structure can be further refined by Hall-Petch formula [32]: *σy* = *σ*0 + *kd* − 1/2; the fine crystallization can effectively enhance the mechanical properties. The magnesium alloy is packed in hexagons; the fine crystallization strengthening effect is much larger than the face of the cubic aluminum alloy. Ding et al. [28] studied the Zr, Sr in Mg-Sr-Zr alloy of nontoxic side on the human metabolism and the best content on the basis of the achievements of scholars. Sn and Ca were also essential elements of the human body. This study preliminarily analyzed the Mg-Ca alloy and added elements which had good biocompatibility. The microstructure, mechanical properties, and corrosion resistance of the magnesium alloy were studied by adding the various amounts of control elements, and the experimental data were provided for the study of corrosion-resistant biocompatible magnesium alloy.

## 2. Experimental Method

### 2.1. Material Preparation

For comparison, 10 mm thick plates of pure Mg (99.99%) were used for a variety of performance tests.

**Figure 1:**
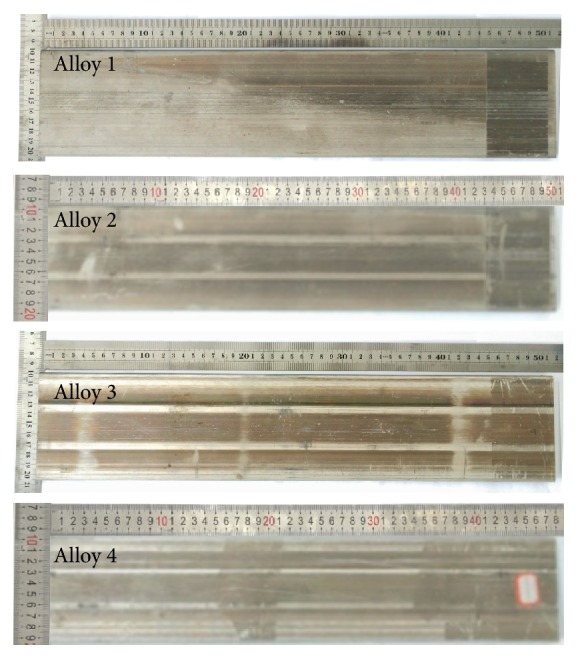
Alloy 1, Alloy 2, Alloy 3, and Alloy 4 after rolling.

Four specimens (Alloy 1, Alloy 2, Alloy 3, and Alloy 4) were prepared by using pieces of pure Mg (99.99%), Mg-30Sr alloy, Mg-10Ca alloy, and pure Sn and Mg-30Zr alloy (Figure 1). The 10 mm thick rolling plates of Alloy 1, Alloy 2, Alloy 3, and Alloy 4 were prepared by the following method.

(1) Pieces of the pure Mg, pure Sn, and the alloys were melted in a crucible resistance furnace under the protection of 0.01% CO_2_ + SF_6_. Pieces of Mg-30Sr alloy should be added in the end because they were easy to be burned.

(2) Then 0.1% CCl_4_ slag was added in the melts and was heated to 720°C for the degassing and grain refining purpose. When the alloy was completely melted and stirred at 720°C for 5 minutes, then the temperature was raised to 750°C for 20 minutes and the furnace was turned down. After the furnace temperature dropped to 720°C, the alloy liquid was poured into a steel mold (H13 die steel) which had been preheated to 200°C to obtain an ingot. Before pouring, the mold was coated with a thin layer of releasing agent for casting.

(3) After air cooling, the ingot was heated to 300°C and placed on a hot rolling mill to be rolled in a mold with dimensions of *φ*80 mm in inner diameter, 20 mm in wall thickness, and 500 mm in height and rolled to 10 mm thickness by one-step molding at a rolling ratio of 16 : 1. Before extrusion, the magnesium alloy bar was kept in a holding furnace at 300°C for about 30 minutes to ensure that the bar was completely heated and the extruded cylinder was heated to 300°C before extrusion. Magnesium alloy hot extrusion die coating lubricant was applied.

### 2.2. Microstructure Characteristics

The alloy samples were cut on the rolled alloy specimens (Table 1) and in parallel direction with rolling. Five samples (pure Mg, Alloy 1, Alloy 2, Alloy 3, and Alloy 4) were ground from 400 grit to 2000 grit with SiC sandpaper and polished on a PG-2 metallographic sample polishing machine and then etched with corrosive (distilled water 80 mL, picric acid 5 g, and acetic acid 15 mL). The pure Mg and four as-cast alloys' microstructure was observed by LEICA DM 2500M optical microscope and the EMPYREAN XRD were used to analyze the phase with a Cu K*α* radiation over the range 20° ⩽ 2*θ* ⩽ 80° with an accelerating voltage of 40 kV and a current of 250 mV at room temperature. The samples were scanned at 2°/min. The morphology of the alloy was observed by MLA 650F SEM, and the composition was analyzed by EDS.

### 2.3. Mechanical Performance Test

The tensile test samples and compression test samples were cut from rolled Mg alloy specimens in parallel direction of rolling. For comparison, 3 mm thick plates of pure Mg (99.99%) were used for a variety of performance tests.

The tensile test specimens (the gauge part was 50 mm in length, 12.5 mm in width, and 3 mm in thickness) and compression test specimens (8 mm high and 5 mm in diameter) prepared according to ASTM E8 [29], E8M-13a [30], and ASTM E9-89a [31] were stretched and compressed, respectively, using a W-100 microcomputer controlled electronic universal testing machine at a draw rate of 0.5 mm/min and a compression test rate of 0.01 mm/min. Three sets of tensile test specimens and 3 sets of compression test specimens were tested, respectively, and the average values were calculated.

### 2.4. Electrochemical Tests

Alloy samples cut into 1 cm *∗* 1 cm *∗* 1 cm pieces were ground from 400 grit to 2000 grit with SiC sandpaper and polished on a PG-2 metallographic sample polishing machine and then placed in Hank's physiological solution at a constant temperature of 37°C (Hank's solution composition: 8.01 g/L NaCl, 0.40 g/L KCl, 0.19 g/L CaCl_2_, 0.051 g/L Na_2_HPO_4_·H_2_O, 0.055 g/L KH_2_PO_4_, 0.353 g/L NaHCO_3_, and 0.346 g/L MgSO_4_·7H_2_O) to evaluate the corrosion rate with hydrogen evolution. The epoxy resin protection was made on specimens and the specimens were only 0.5 cm^2^ exposed to the solution. The CS310 multifunction electrochemical workstation was applied with calomel electrode as the reference electrode; Pt was applied as the auxiliary electrode of the three-electrode system to measure the open circuit potential. The scanning speed was set at 1 mv/s to measure the polarization curve. The dynamic potential scanning range of −0.5 V to 1.5 V and Tafel traditional method were used for fitting.

### 2.5. Cell Culture

Cells for CVR test were cultured in low-limit basal medium (MEM) (Gibco, Invitrogen, Mulgrave, VIC, Australia) (Barwon Biomedical Research, Geelong Hospital, Victoria, Australia) with osteoblast characteristics. Other reagents include 10% fetal bovine serum (Bovogen (Sigma-Aldrich, Castle Hill, NSW, Australia), 10,000 units/mL penicillin-10000 *μ*g/mL streptomycin (Gibco) and 0.4% amphostat B (In Vitro Technologies), 1% nonessential amino acids (Sigma-Aldrich, Castle Hill, NSW, Australia), Auckland, New Zealand) and were cultured in a humidified air atmosphere at 37°C and 5% CO_2_. The medium was replaced every 3 days.

### 2.6. Cell Viability Ratio (CVR) Test

The Mg-Ca alloy samples of *φ*8 × 2 mm were sterilized at 180°C for 2 h and then placed into the cell culture plate with control samples, 5 × 103 cells were seeded on each sample for 7 days, and the biocompatibility was evaluated by the international standard ISO10993-5 [33].

## 3. Results and Discussion

### 3.1. Composition and Microstructure

Rolled alloy specimens are listed in Table 1. Three small samples (1 cm *∗* 1 cm *∗* 10 cm) were cut at three different locations on the specimens. Elemental compositions of three samples were tested by S4 PIONEER XRF and the results listed in Table 1 were the average content.

Chemical analysis of Alloy 1 showed that average content of Zr was 3.97, and 3 samples were 3.88, 4.10, and 3.95, respectively. Average content of Zr in Alloy 2 was 1.48, and 3 samples were 1.48, 1.39, and 1.58, respectively. Average content of Zr in Alloy 3 was 1.37, and 3 samples were 1.29, 1.36, and 1.47, respectively. Hence, there was macrosegregation of the elements in Alloy 1, Alloy 2, and Alloy 3.

Chemical analysis showed that there was no macrosegregation of the elements in Alloy 4. Average content of Sn in Alloy 4 was 0.48, and 3 samples were 0.47, 0.48, and 0.48, respectively.

**Figure 2:**
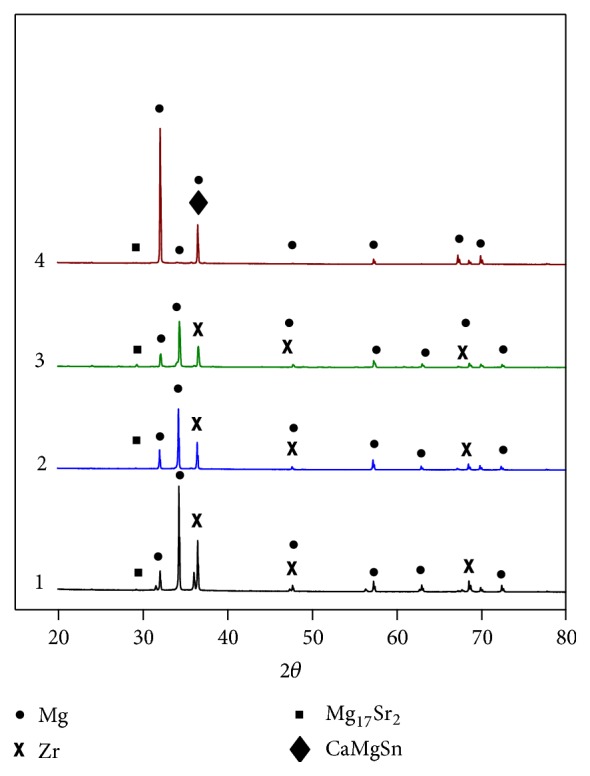
XRD patterns of rolled Alloy 1, Alloy 2, Alloy 3, and Alloy 4.

Phase compositions of four magnesium alloys were determined by XRD (Figure 2). It could be seen from the figure that Alloy 1, Alloy 2, and Alloy 3 were mainly composed of *α*-Mg matrix, Zr, and intermetallic compound Mg_17_Sr_2_. After adding Sn, CaMgSn phase was formed beside *α*-Mg matrix and Mg_17_Sr_2_, and the diffraction angle of the strongest peak showed deviation, which may be due to the rolling of as-cast alloy and different additives.

**Figure 3:**
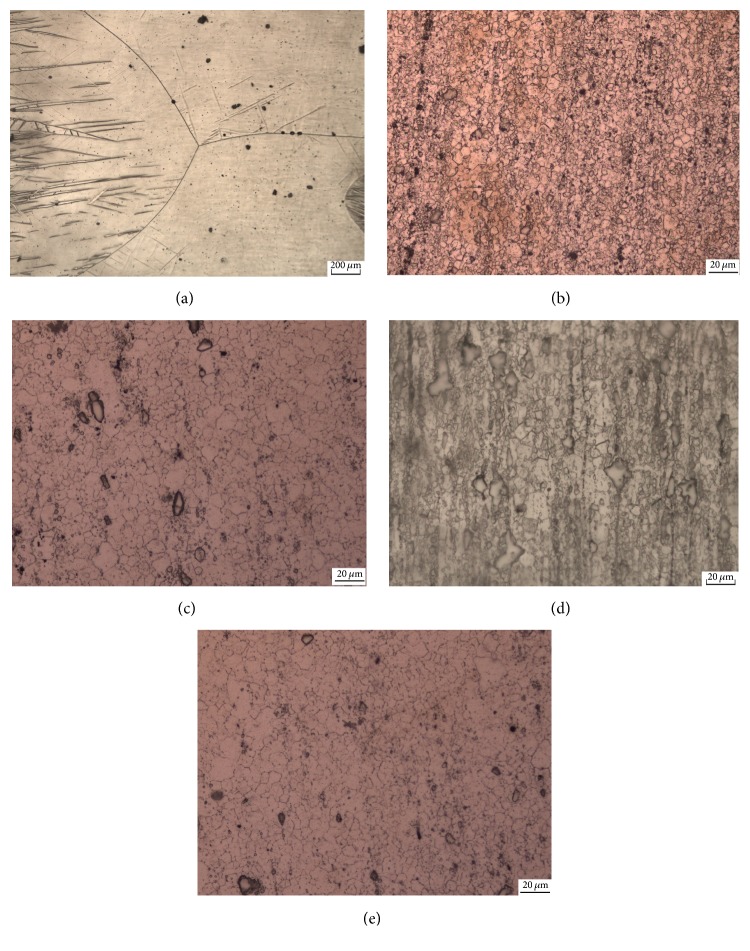
Microstructures of the cross sections of rolled alloys: (a) pure Mg; (b) Alloy 1; (c) Alloy 2; (d) Alloy 3; (e) Alloy 4.

Figure 3 shows the cross-section metallographic photographs of pure Mg and four groups of rolled magnesium alloy (Alloy 1, Alloy 2, Alloy 3, and Alloy 4). It can be seen from the figure that, compared to pure Mg in Figure 3(a), the grain sizes of magnesium alloy had been refined after rolling, which might be caused by alloying and alloy dynamic recrystallization. The black points should be pure *α*-Zr particles (the maximum solid solubility in the magnesium alloy is 0.6%), and the XRD spectrum of Figure 2 shows that Alloy 1 had a strong Zr prime peak. Although the average grain sizes of Figure 3(e) were 9.7 *μ*m according to linear intercept method in ASTM E112 [34], which was significantly larger than that of Figure 3(b), 5.36 *μ*m, Figure 3(c), 6.71 *μ*m, and Figure 3(d), 6.16 *μ*m, it can be seen that the distribution of the components in Figure 3(e) was relatively uniform. After being rolled, the grains in the alloys were found to be stretched and there were some incomplete recrystallization and recovery zones. In addition, the cross sections were formed by equiaxed grains. The dynamic recrystallization after rolling was secondarily refined to that of the grains.

**Figure 4:**
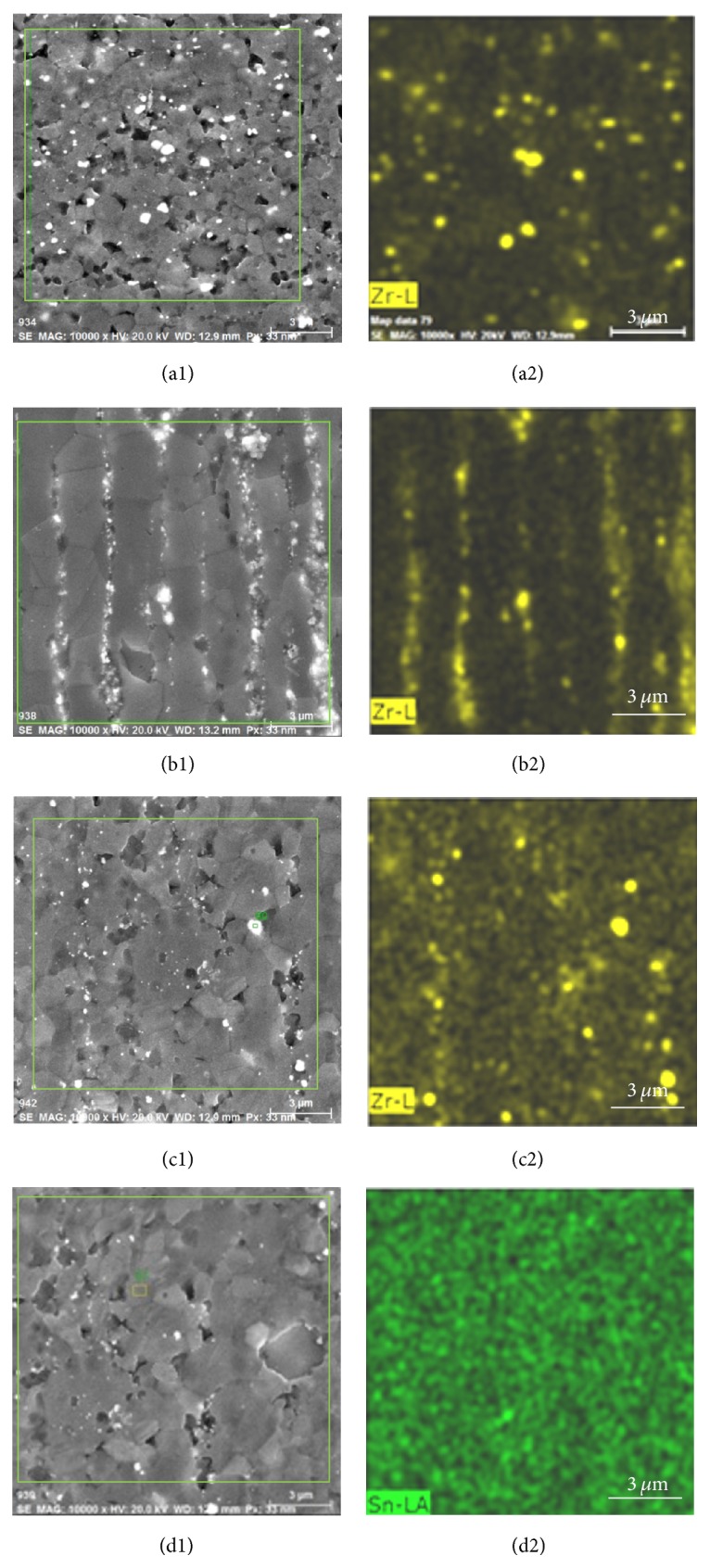
Images of (a1) SEM of Alloy 1, (a2) Zr EDS pattern of Alloy 1, (b1) SEM of Alloy 2, (b2) Zr EDS pattern of Alloy 2, (c1) SEM of Alloy 3, (c2) Zr EDS pattern of Alloy 3, (d1) SEM of Alloy 4, and (d2) Sn EDS pattern of Alloy 4.

Figure 4 shows SEM, Zr EDS, and Sn EDS images of Alloy 1, Alloy 2, Alloy 3, and Alloy 4, respectively. In Figures 4(a2), 4(b2), and 4(c2), it can be seen that the irregular circular white areas were *α*-Zr-rich segregation areas, and the distribution of Zr was not very uniform. The crystallization of Alloy 1, Alloy 2, and Alloy 3 was more refined than that of pure magnesium (Figure 3) because a small amount of Zr was co-solid-solved into the *α*-Mg matrix.

The uniformly distributed Sn element was found by EDS mapping in Figure 4(d2). Therefore, the crystallization of Alloy 4 was more refined than that of pure magnesium and Alloy 1, Alloy 2, and Alloy 3 (Figure 3).

### 3.2. Mechanical Properties

**Figure 5:**
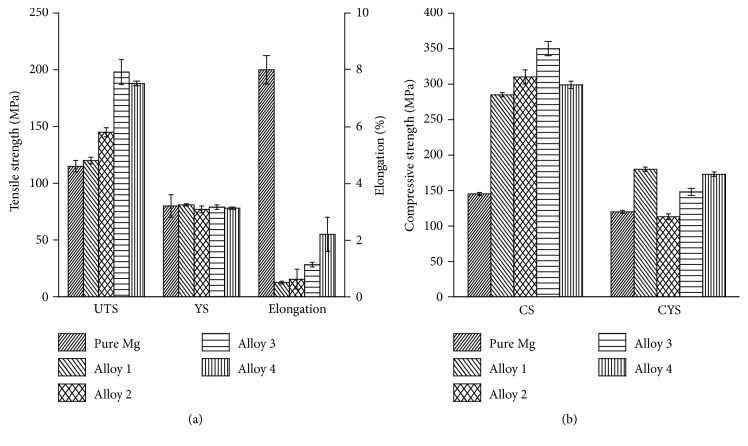
Tensile strength and elongation (a) and compressive strength (b) of pure Mg and Alloy 1, Alloy 2, Alloy 3, and Alloy 4.

The mechanical properties of pure Mg and four alloys are shown in Figure 5. The comprehensive mechanical properties of four rolling alloys were much higher than those of pure Mg, which may be due to factors such as solid solution and second-phase strengthening of Zr, Ca and work hardening of Mg_2_Sn, Mg_17_Sr_2_. The compressive strength of Alloy 3 was relatively better than others. Mg_17_Sr_2_ phases in alloys could refine crystalline grains. The elongation of Alloy 4 was higher than that of 3 alloys, demonstrating that the Mg_17_Sr_2_ phases in the alloy homogenized the distribution of CaMgZn phases and contributed to the alloy elongation.

Compressive strength of the alloys was more than twice as high as that of pure magnesium. This was because a large number of second-phase particles (Mg_2_Sn, Mg_17_Sr_2_) greatly improve the compressive strength of the alloys.

### 3.3. Corrosion Resistance

**Figure 6:**
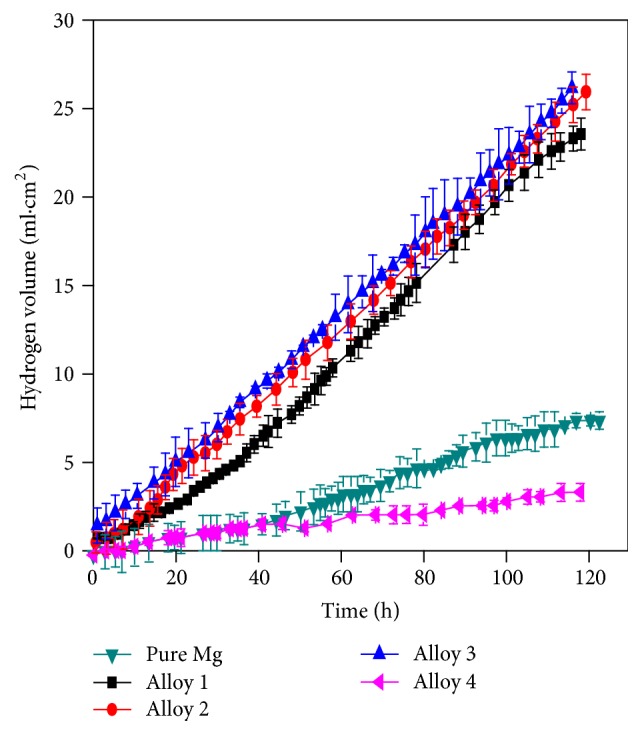
Variations of hydrogen evolution volumes of pure Mg and Mg alloys in Hank's solution with immersion time.

As shown in Figure 6, pure Mg and Mg alloys 1, 2, 3, and 4 were immersed in Hank's solution at 37°C to evaluate the amount of hydrogen evolution with immersion time. It can be seen from the figure that the curve of Alloy 4 was close to a straight line in the 120-hour experimental period, indicating that Alloy 4 basically remained in a relatively consistent dissolution rate. The other four groups of alloys and pure Mg were initially in rapid hydrogen evolution rate; then the rate began to decline as time went on. It can be seen from the figure that only the hydrogen deposition amount of Alloy 4 was close to that of pure Mg and was more resistant to corrosion than pure Mg. Corrosion resistance of Alloy 1, Alloy 2, and Alloy 3 was relatively closer, which was inferior to that of pure Mg. According to the daily precipitation of hydrogen, from the formula ph = 2.279Vh [35], the calculation result of the corrosion rate of the alloys per year is shown in Table 2.

**Figure 7:**
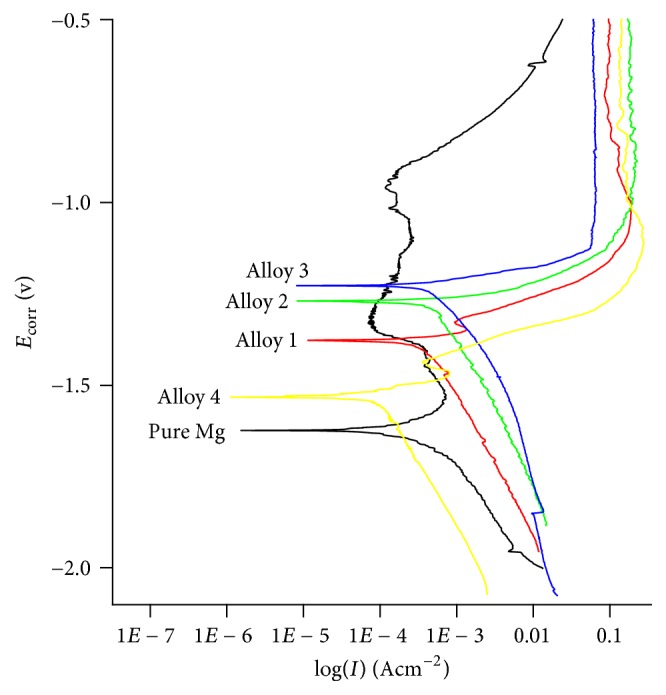
Polarization curves in Hank's solution of rolled pure Mg and Mg alloys.

Figure 7 is the polarization curve of pure Mg and Mg Alloy 1, Alloy 2, Alloy 3, and Alloy 4 in Hank's solution at 37°C. It can be seen from the figure that, in the cathode region, the corrosion current of Alloy 4 was much smaller than that of pure Mg and other 3 alloys at the same potential, and the corrosion current of the other 3 alloys was larger than that of pure Mg, indicating that the corrosion resistance of Alloy 4 was most optimal, and other alloys were less corrosion-resistive than pure Mg, which was accordant with the results measured by the hydrogen evolution method. In the anode area, it was apparent that pure Mg and Alloy 4 had inflection points, respectively, indicating that corrosion-resistant passivation films were formed, thereby reducing the corrosion rate, while inflection points in the other three groups of alloys were not particularly significant and are basically close to active dissolution, especially Alloy 3. The results were fitted by Tafel traditional method, and the corrosion potential and current density are shown in Table 4.

Table 3 shows the corrosion potential (*E*_corr_) and corrosion current densities (*I*_corr_) of phosphate coatings. Electrochemical parameters showed results correspond to potentiodynamic polarization curves of the alloys shown in Figure 7. *E*_corr_ of Alloy 1, Alloy 2, Alloy 3, and Alloy 4 showed lower *E*_corr_ than that of pure Mg. The Mg-0.5Ca-1Sr-0.5Sn alloy demonstrated lowest corrosion current and corrosion rate in electrochemical test; *I*_corr_ was 25% of that of pure magnesium and showed delayed corrosion in Hank's solution.

**Figure 8:**
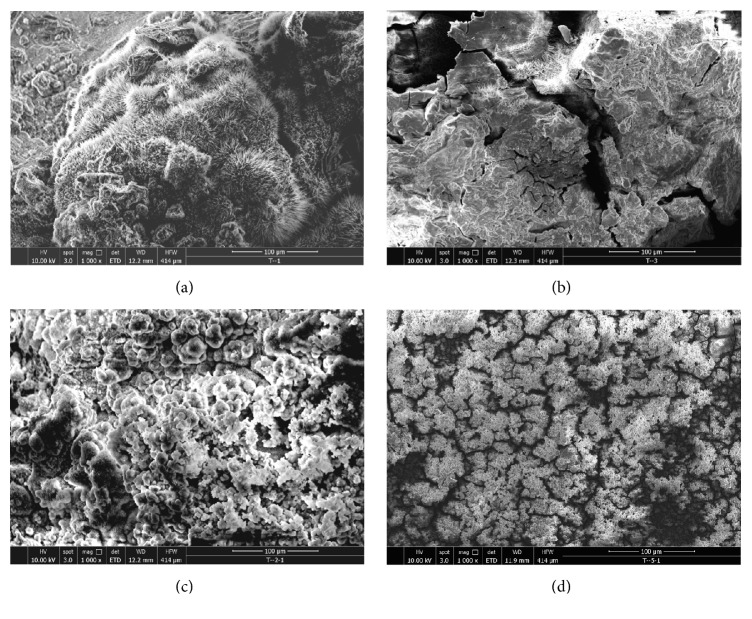
The SEM images of the alloys immersed in Hank's solution after 120 h: (a) Alloy 1; (b) Alloy 2; (c) Alloy 3; (d) Alloy 4.

Figure 8 shows SEM images of Alloy 1, Alloy 2, Alloy 3, and Alloy 4 after being immersed in Hank's solution for 120 h and washing away the corrosion product. Corrosion microcracks could be seen and they were not shown in the original morphologies on the surface of Alloy 1 and Alloy 2 samples. There were severe bulges because *α*-Zr in the Zr-rich region acted as cathode in corrosion galvanic and accelerated the corrosion in the process of corrosion. That is the reason why the corrosion resistance of Alloy 1 and Alloy 2 was poor. The original morphology on the surface of Alloy 3 samples was more complete but there were bulges because of electrochemistry corrosion.

The original morphology on the surface of Alloy 3 samples was more complete without corrosion. Mg_17_Sn_2_ compounds made CaMgSn homogeneous and corrosion was blocked.

### 3.4. Biocompatibility Assessment

Table 4 shows the cell viability ratio (CVR) values used for assessing the in vitro cytotoxicities of the Mg-Ca alloys, compared to control group. The cell viability ratio (CVR) was calculated using the equation given by [36]:

(1) $$\begin{array}{c} \text{CVR}=\frac{\left(\text{viable cell count in experimental extract}\right)}{\left(\text{viable cell count in control extract}\right)}. \end{array}$$

**Figure 9:**
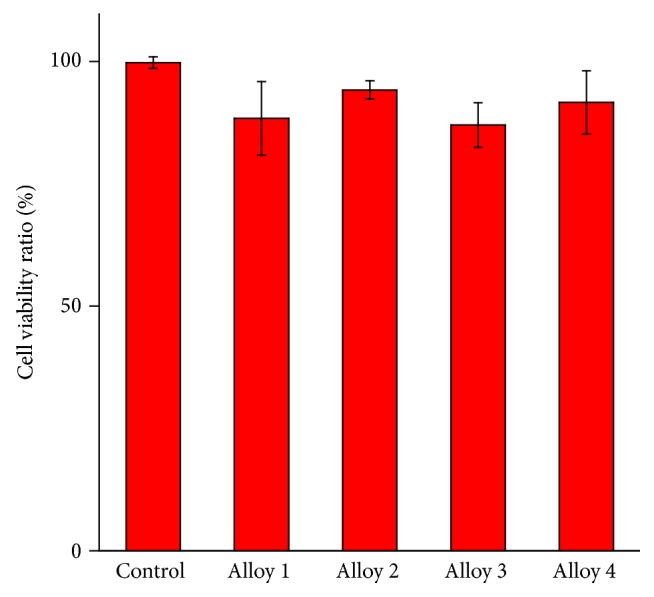
Cell viability ratio of alloys after 24 h extract cell seed for 1 day and then cell cultures for 5 days.

As can be seen from Figure 9, the cell viability ratio (CVR) values of Alloy 1, Alloy 2, Alloy 3, and Alloy 4 were 0.89, 0.94, 0.87, and 0.92, respectively. They were close to the results of the control group, where Alloy 2 and Alloy 4 showed slightly higher CVR in the materials evaluated. Therefore, it can be inferred that Alloy 2 and Alloy 4 had better cell compatibility.

## 4. Conclusion

A new series of Mg-Ca alloys including Mg-0.5Ca-1Sr-4Zr, Mg-0.5Ca-1Sr-1.5Zr, Mg-0.5Ca-3Sr-1.5Zr, and Mg-0.5Ca-1Sr-0.5Sn were designed using the vacuum smelting, gravity casting, and hot rolling approaches. The microstructures, mechanical properties, and cytocompatibility of the Mg-Ca alloys were investigated. The following conclusions can be drawn from this study.

(1) The alloys were composed of *α*-Mg, *α*-Zr, Mg_12_Sr_7_, and CaMgSn, and their grain sizes were dramatically refined compared with that of pure Mg, which were characterized by optical microscopy, XRD, and EDS.

(2) The comprehensive mechanical properties of alloys containing Zr were greatly improved; Mg-0.5Ca-3Sr-1.5Zr alloy obtained the highest tensile strength of approximately 200 MPa and highest compression strength at 350 MPa. But the corrosion resistance of Mg-Ca-Sr-Zr alloys was not as good as that of pure Mg. The alloy showed balanced mechanical properties, with tensile strength of 180 Mpa and compressive strength of 340 Mpa, which was a bit lower than Mg-0.5Ca-3Sr-1.5Zr alloy, but superior to pure Mg.

(3) The Mg-0.5Ca-1Sr-0.5Sn alloy released the least hydrogen in Hank's solution, which was lower than that of pure magnesium. Electrochemical test results in Hank's solution further showed that the Mg-0.5Ca-1Sr-0.5Sn alloy had delayed corrosion and lowest *I*_corr_ which was 25% of that of pure magnesium.

(4) The Mg-Ca alloys exhibited excellent cytocompatibility on osteoblast-like cells (SaOS_2_).

## Figures and Tables

**Table 1:** The specimens and composition by XRF (mass%).

Rolled alloy specimen	Designed content	Actual content by XRF	Impurity content by XRF (wt%)
Alloy 1	Mg-0.5Ca-1Sr-4Zr	Mg-0.45Ca-0.74Sr-3.99Zr	0.016% Fe, Si, Cu, Ni undetected.
Alloy 2	Mg-0.5Ca-1Sr-1.5Zr	Mg-0.55Ca-1.07Sr-1.49Zr	0.009% Fe, 0.11% Si, Cu, Ni undetected.
Alloy 3	Mg-0.5Ca-3.5Sr-1.5Zr	Mg-0.54Ca-3.37Sr-1.37Zr	0.017% Fe, 0.083% Si, Cu, Ni undetected.
Alloy 4	Mg-0.5Ca-1Sr-0.5Sn	Mg-0.43Ca-1.21Sr-0.48Sn	0.020% Fe, 0.12% Si, Cu, Ni undetected.

**Table 2:** Hydrogen evolution and corrosion rates of pure Mg and Mg alloys in Hank's solution.

Samples	H_2_ evolution rate mL/(cm^2^ day)	Corrosion rate^(1)^ mm/year
Pure Mg	1.43	3.26
Alloy 1	4.79	10.92
Alloy 2	5.14	11.71
Alloy 3	5.32	12.11
Alloy 4	0.68	1.56

^(1)^Corrosion rate mm/year was calculated with H_2_ production rate.

**Table 3:** Electrochemical parameters of pure Mg and Mg alloys.

Sample code	*E*_corr_ (V)	*I*_corr_ (mA/cm^2^)	Corrosion rate (mm/year)
Pure Mg	−1.38	0.48	10.39
Alloy 1	−1.26	0.72	15.63
Alloy 2	−1.21	0.74	16.12
Alloy 3	−1.48	3.09	67.06
Alloy 4	−1.55	0.11	2.42

**Table 4:** Cell viability ratio of alloys after 24 h extract cell seed for 1 day and then cell cultures for 5 days.

	Viability (% of control)			Average (%)	STD
	1	2	3		
Control	99.99	98.54912	101.4509	100%	1.18
Alloy 1	80.05	91.2	94.45	88.57	7.55
Alloy 2	94.7	92.41	96.1	94.40	1.86
Alloy 3	91.7154	87.32	82.6	87.21	4.56
Alloy 4	84.4	96.2	94.89	91.83	6.47
